# Association Between Peritoneal Dialysis-Associated Peritonitis and the Risk of All-Cause Mortality and Cardiovascular Death: A Time-Matched Retrospective Cohort Study

**DOI:** 10.3390/medsci13040249

**Published:** 2025-10-30

**Authors:** Surapon Nochaiwong, Kajohnsak Noppakun, Manish M. Sood, Kednapa Thavorn, Greg A. Knoll, Chidchanok Ruengorn, Apichat Tantraworasin

**Affiliations:** 1 Center for Clinical Epidemiology and Clinical Statistics, Faculty of Medicine, Chiang Mai University, Chiang Mai 50200, Thailand; surapon.nochaiwong@cmu.ac.th; 2 Department of Pharmaceutical Care, Faculty of Pharmacy, Chiang Mai University, Chiang Mai 50200, Thailand; chidchanok.r@cmu.ac.th; 3 Pharmacoepidemiology and Statistics Research Center (PESRC), Chiang Mai University, Chiang Mai 50200, Thailand; kajohnsak.noppakun@cmu.ac.th (K.N.); kthavorn@ohri.ca (K.T.); 4 Division of Nephrology, Department of Internal Medicine, Faculty of Medicine, Chiang Mai University, Chiang Mai 50200, Thailand; 5 Division of Nephrology, Department of Medicine, University of Ottawa, Ottawa, ON K1H 8L6, Canada; msood@toh.ca (M.M.S.); gknoll@toh.ca (G.A.K.); 6 Institute of Clinical and Evaluative Sciences, ICES uOttawa, Ottawa, ON K1Y 4E9, Canada; 7 Ottawa Hospital Research Institute, Ottawa Hospital, Ottawa, ON K1H 8L6, Canada; 8 School of Epidemiology and Public Health, Faculty of Medicine, University of Ottawa, Ottawa, ON K1G 5Z3, Canada; 9 Department of Surgery, Faculty of Medicine, Chiang Mai University, Chiang Mai 50200, Thailand; 10 Clinical Surgical Research Center, Chiang Mai University, Chiang Mai 50200, Thailand

**Keywords:** cardiovascular death, infection, mortality, peritoneal dialysis, peritonitis

## Abstract

**Background/Objectives**: Although peritoneal dialysis (PD) practices have improved over the past decades, limited evidence exists on all-cause mortality and cardiovascular death following PD-associated peritonitis. This study aimed to investigate the association between PD-associated peritonitis and the risk of all-cause mortality and cardiovascular death. **Methods**: This multicenter, retrospective cohort study included adult patients who newly initiated PD between 1 January 2006 and 31 December 2020, with follow-up through 30 September 2022. Patients were matched 1:1 by time from PD initiation to index date (the occurrence date of PD-associated peritonitis for the exposure group and the corresponding matched time on PD for the non-exposure group [individuals without any peritonitis event]), age, and sex. Multivariable Cox proportional hazards models with shared frailty correction and competing risk models were used to estimate hazard ratio (HR) and subdistribution hazard ratio (SHR), respectively. Subgroup analyses were conducted by age, sex, PD modality, and comorbid conditions. **Results**: The cohort included 1510 matched pairs (total sample, 3020; mean age [SD], 58.6 [14.2] years; 1618 males [53.6%]), with a median follow-up of 5.6 years. After adjusting for sociodemographic, PD, and clinical characteristics and laboratory profiles, patients with any PD-associated peritonitis episode had significantly higher risk of all-cause mortality (HR, 2.17 [1.78–2.66], *p* < 0.001; SHR, 2.00 [1.74–2.29], *p* < 0.001) and cardiovascular death (HR, 2.90 [2.05–4.59], *p* < 0.001; SHR, 2.25 [1.66–3.05], *p* < 0.001) compared to those without PD-associated peritonitis. Subgroup analyses revealed no significant interactions (all *p* values for interaction > 0.05). **Conclusions**: PD-associated peritonitis was independently associated with substantially increased risk of all-cause and cardiovascular mortality among patients undergoing PD. These findings support the need for targeted interventions and clinical strategies aimed at reducing adverse outcomes following PD-associated peritonitis.

## 1. Introduction

Peritoneal dialysis (PD) represents a prioritized form of kidney replacement therapy, particularly in resource-limited settings [1]. Globally, PD was available in 79% of countries, with an estimated median prevalence of 21.0 per million population [2]. With respect to patients’ autonomy, PD offers flexibility for end-stage kidney disease (ESKD) patients to perform a home-based approach [3,4]. Compared with hemodialysis, PD also offers several potential advantages, including lower health care utilization, lower burden on trained staff and health care management, better physical outcomes and quality of life, and better options during the transition to kidney transplant [1,3,5].

From the health care professional and patient/caregiver perspectives, PD-associated peritonitis was recognized as a critically important outcome for PD management [6]. Although PD practices have been improved over the past decades, PD-associated peritonitis remains a serious adverse outcome in the PD population [1,7,8]. It is well established that peritonitis leads to peritoneal membrane deterioration and malfunctions, which limit the long-term PD utilization [4]. Indeed, the risk of adverse outcomes, including both mechanical and non-mechanical complications, may increase over time following an episode of PD-associated peritonitis [4,7,8,9]. Not only the acute infectious-related mortality, but also long-term non-infectious mortality risk, via chronic inflammation and peritoneal membrane function deteriorations, have been postulated in addition to traditional cardiovascular factors [10,11]. In 2022, approximately 68.9% of the United States adult PD population was reported to have any cardiovascular diseases [12]. Moreover, the commonest cause-specific deaths among ESKD patients treated with PD were attributed to cardiovascular disease, ranging from 24% to 42% [13,14]. Unfortunately, there are no specific guidelines or treatment recommendations that have been established to reduce the risk of mortality or adverse cardiovascular outcomes following PD-associated peritonitis events [8,15]. In addition, to date, strategies for the prevention of PD-related infections have yielded inconsistent results in reducing the mortality burden in this population [8,15,16,17].

To the best of our knowledge, existing studies with varied approaches of analysis have provided valuable insights into the risk of death and PD-associated peritonitis [10,11,18,19,20]. However, previous studies have not fully accounted for sociodemographic diversity, clinical characteristics, and key laboratory variables, which are subject to residual confounders [10,11,18,20]. Existing studies have presumed an observation period (i.e., time-attributable risk) from the initiation of PD to death or the censoring date in all patients or have limited the window period to 3 to 9 months preceding death [10,11,18,19,20]. This limited statistical analysis is primarily related to immortal time lead bias and the brevity and intermittent nature of the at-risk period. As a result, existing estimates of the risk of death after the occurrence of PD-associated peritonitis may not accurately reflect long-term mortality, including cardiovascular death among ESKD patients undergoing PD treatment.

To address these knowledge gaps and account for immortal time lead bias before the risk of peritonitis episode, we leveraged a time-matched pair retrospective cohort design of individuals with and without any PD-associated peritonitis using patient-level data from the integrated health care system to investigate the risk of all-cause mortality and cardiovascular death following the episode of PD-associated peritonitis.

## 2. Methods

### 2.1. Study Design, Data Sources, and Eligible Patients

We conducted a multicenter, time-matched retrospective cohort study based on the Thai Renal Outcomes Research-Peritoneal Dialysis database. All data were based on linked datasets that consecutively enrolled ESKD patients who newly initiated PD in three centers in Chiang Mai province. This database represents one of the largest PD centers in Northern Thailand and pro-vides PD programs and training under the “PD First Policy” in Thailand. Moreover, the database has been used to conduct Thai PD studies, and the details are described elsewhere [7,21,22]. In this study, individuals with any PD-associated with peritonitis were matched with individuals without peritonitis (1:1). We extracted and included the information as follows: (1) electronic health records, a claim database that provides outpatient and inpatient information; (2) the PD Patient Care Database that contains individual patient-level sociodemographic and PD treatment care; and (3) the Laboratory Support System extract that provides claims laboratory information.

Eligible patients for this study included adult ESKD patients aged 18 years or older who newly initiated PD between 1 January 2006, and 31 December 2020, and were followed until 30 September 2022. We excluded patients who had: (1) initiated PD treatment for acute kidney injury, (2) treated with PD for less than 90 days, (3) incomplete follow-up information, and relevant data on PD-associated peritonitis.

Ethical approval for the study was obtained from the institutional review boards of the Faculty of Medicine, Chiang Mai University (No. 428/2021; approval date: 5 October 2021) and Nakornping Hospital (No. 076/64; approval date: 19 July 2021), Chiang Mai, Thailand. Informed consent was waived due to the nature of the study design, and all patients’ information was de-identified and anonymized to protect patient confidentiality. We reported in accordance with the Strengthening the Reporting of Observational Studies in Epidemiology guidelines [23].

### 2.2. Exposure, Non-Exposure, and Covariates

The exposure group of interest consisted of individuals diagnosed with PD-associated peritonitis according to the current 2022 International Society for Peritoneal Dialysis (ISPD) guidelines [8]. The occurrence of PD-associated peritonitis was determined as the presence of at least 2 of the following: (1) clinical features consistent with peritonitis (i.e., abdominal pain and/or cloudy dialysis effluent); (2) dialysis effluent white blood cell count >100 cells/µL, with >50% polymorphonuclear leukocytes; and (3) positive dialysate effluent culture [8].

Regarding a matched-pairs design based on time on PD (within 1 month), age (within 1 year), and sex, each patient who experienced PD-associated peritonitis was matched only once to a patient without any peritonitis event. The date of diagnosis of PD-associated peritonitis was defined as the index date (inception time) of this study cohort. Based on age, sex, and time-matched, the risk window period started from the index date (the occurrence date of PD-associated peritonitis for the exposure group and the corresponding matched time on PD for the non-exposure group [individuals without peritonitis event). For instance, based on study eligibility criteria, if an individual had the occurrence of peritonitis at 1 year after the PD initiation, then the matched control for this case was randomly selected among individuals without any PD-associated peritonitis during the follow-up period, in which the index date was identified according to the pair-matched case (1 year time-matched).

Covariate information was derived within the prelude period (between the PD initiation date [post-break-in period and the date of diagnosis with PD-associated peritonitis] for the exposure group and the time-matched index date for the non-exposure group). These variables included sociodemographic (i.e., age, sex, body mass index [BMI], marital status, work status, educational level, smoking and alcohol drinking status, reimbursement scheme, and living distance from PD center), PD and clinical characteristics (i.e., era of PD initiation, urgent-start PD [based on catheter break-in period ≤14 days], estimated glomerular filtration rate [eGFR] at PD initiation, PD modality [continuous ambulatory PD; CAPD and automated PD; APD], etiology of ESKD, Charlson comorbidity index, comorbid conditions [coronary artery disease, chronic heart failure, cerebrovascular disease, diabetes mellitus], residual kidney function [defined as urine output ≥200 mL/day at PD initiation]), and blood laboratory profiles (creatinine, urea nitrogen, albumin, hemoglobin, ferritin, transferrin saturation, sodium, potassium, bicarbonate, calcium, phosphorus, intact parathyroid hormone, and alkaline phosphatase).

### 2.3. Outcomes

The outcomes of interest were all-cause mortality and cardiovascular death. Mortality outcome was ascertained from the PD Patient Care Database, which provides details on long-term PD care administrative data, and coordinated with the National Health Security Office records, which manage universal health coverage for Thai citizens. All-cause mortality was defined as deaths due to any cause. Cardiovascular events that were attributed as a cause of death (i.e., coronary artery diseases, congestive heart failure, stroke, or systemic embolism) were identified from primary diagnoses assigned during hospital admissions based on the International Classification of Diseases, 10th revision, captured through electronic health records, death-certified records, and medical chart review. The outcomes of interest were identified after the index date, and follow-up was censored at the study end date (30 September 2022) or death or transfer to other dialysis centers, loss to follow-up, or discontinuation of PD (kidney transplantation/technical failure [switched to hemodialysis]), whichever comes first.

### 2.4. Statistical Analyses

To ensure rigorous data quality, two independent investigators (SN and KN) reviewed all potentially eligible patients against the 2022 ISPD guidelines [8] and verified the cause of death. Based on previous reports, the least differential adjusted hazard ratio (HR) for cardiovascular death among existing studies was 1.31, with a median 2.6-year survival rate of 85.5% [11]. To ensure 80% power with a two-sided alpha level of 0.05, a total sample size of 2638 PD patients with 436 expected deaths was required to detect an increased risk of mortality among PD patients with any PD-associated peritonitis compared to individuals without PD-associated peritonitis.

Descriptive data are expressed as the number with percentage and mean with standard deviation (SD) or median with range as appropriate. Based on matched pairs analysis, we employed multivariable Cox proportional hazards models with shared frailty correction to examine the association between the exposure and outcomes of interest [24,25]. The assumption of proportional hazards was explored and tested using Schoenfeld residuals plots. Considering discontinuation of PD (i.e., kidney transplantation or switched to hemodialysis) or non-cardiovascular death as a competing event, we also employed multivariable Fine and Gray subdistribution hazard models [26,27]. A complete case analysis approach was considered due to a low rate of missing data (0.2–0.9%). A series analysis based on consecutive 3 model adjustments were performed as follows: (1) model 1, includes year of PD initiation and center of PD treatment; (2) model 2 includes model 1 plus age, sex, BMI, marital status, employment status, educational level, smoking and alcohol drinking status, reimbursement scheme, living distance from PD center, urgent-start PD, eGFR at PD initiation, PD modality, etiology of ESKD, Charlson comorbidity index, and residual kidney function; and model 3 (full model) includes model 2 plus laboratory results. Collinearity testing was performed for variables included in the model adjustment.

Subgroup analyses were performed to explore the effects of age (<65 vs. ≥65 years), sex (male vs. female), PD modality (APD vs. CAPD), and comorbid conditions (history of coronary artery disease, chronic heart failure, cerebrovascular disease, and diabetes mellitus) on the effect estimates. Moreover, additional analyses were performed to explore the effects of modifiers based on the frequency of PD-associated peritonitis episodes (1 or ≥2 episodes) and the onset of PD-associated peritonitis (early-onset [time-to-first episode ≤3 months] or late-onset [time-to-first episode >3 months]). To address the robustness of the findings, sensitivity analyses were performed, including reanalysis based on Cox proportional hazards models without shared frailty correction and estimating the expected (E)-value to quantify the impact of unmeasured confounders [28]. The effect estimates were presented as HRs and subdistribution hazard ratios (SHRs) with 95% confidence intervals (CIs). All analyses were performed using Stata version 16.0 (College Station, TX, USA). Two-tailed with a *p*-value < 0.05 was considered statistically significant.

## 3. Results

### 3.1. Cohort Description and Patient Characteristics

**Figure 1:**
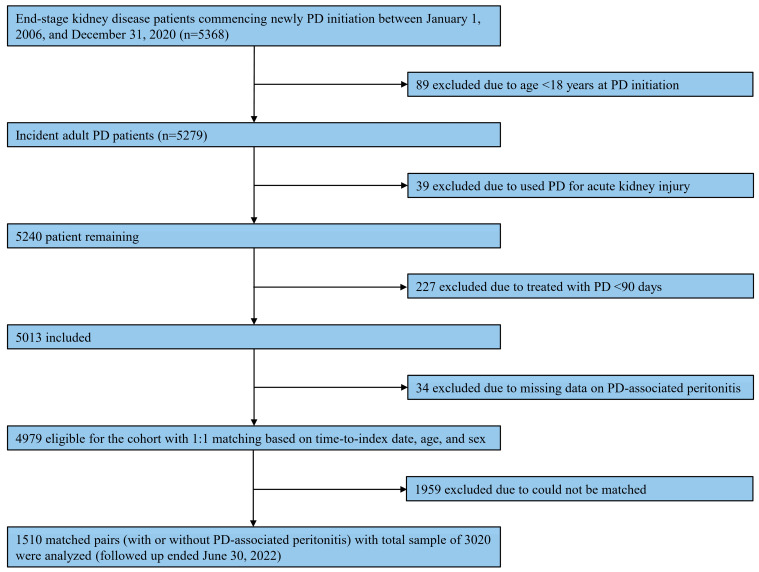
Study flow on the selection of eligible patients. Abbreviations: PD, peritoneal dialysis.

Of 5279 incidents of adult PD patients identified through the database, 1510 matched pairs of patients, with a total sample of 3020, were included in this analysis (Figure 1). Table 1 summarizes patient characteristics according to diagnosis, with or without PD-associated peritonitis, during the observational period. Regarding matching variables, the mean age was 58.6 (SD, 14.2) years, with the majority being male (1618 [53.6%]). The median time-matched was 10.0 (range, 0.1–86.4) months. These matching variables were well-balanced between individuals with and without any PD-associated peritonitis. Most PD patients were treated with CAPD (2615 [86.6%]), and the median Charlson comorbidity index was 5 (range, 2–14). Among individuals with PD-associated peritonitis, the median number of peritonitis episodes was 2 (range, 1–10). Generally, statistically significant differences were observed in patient characteristics between the two groups (Table 1).

### 3.2. Risk of All-Cause Mortality and Cardiovascular Death Following Peritonitis

**Figure 2:**
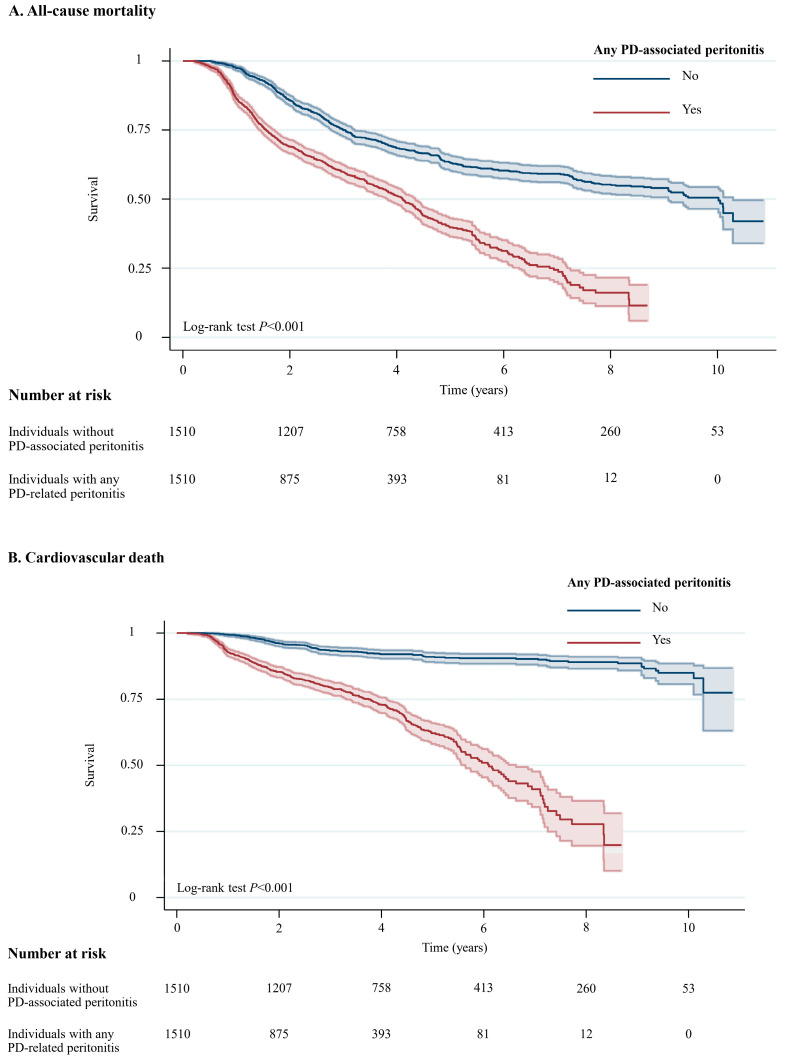
Unadjusted Kaplan–Meier Plots for All-Cause Mortality and Cardiovascular Death Among Individuals with and without PD-Associated Peritonitis. Shaded areas represent 95% CI. Abbreviation: CI, confidence interval; PD, peritoneal dialysis.

A total of 1294 (42.8%) of 3020 patients died during a median follow-up of 5.6 years (10.0 years for individuals without peritonitis and 4.1 years for individuals with any peritonitis episode, with a total of 11,064 patient-years at risk, Table S1 in the Supplementary Materials). Among 1294 individuals who died during the study period, 494 (38.2%) experienced cardiovascular deaths, followed by 308 (23.8%) who died due to infection that was not related to PD, while 101 (7.8%) died due to PD-related infections. For all-cause mortality, Individuals with any PD-associated peritonitis had a higher likelihood of all-cause mortality (crude absolute risk difference = 12.9%) and cardiovascular death (crude absolute risk difference = 18.6%) than those without PD-associated peritonitis (Table S1 in the Supplementary Materials). Figure 2 illustrates unadjusted Kaplan–Meier plots for all-cause mortality (*p* < 0.001) and cardiovascular death (*p* < 0.001).

**Figure 3:**
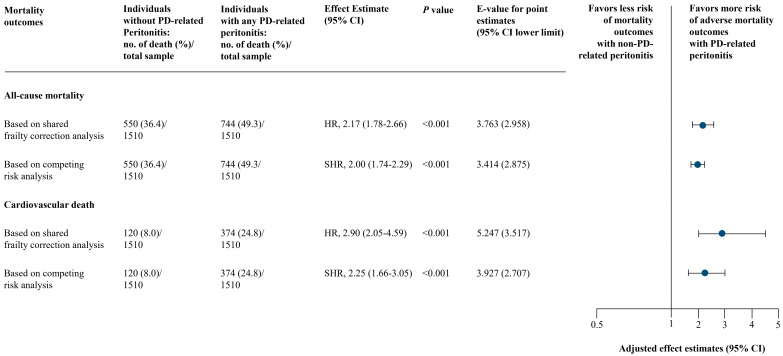
Main Analysis of PD-Associated Peritonitis and Mortality Outcomes. Based on adjusted model 3 (full model) includes: year of PD initiation and center of PD treatment, age, sex, body mass index, marital status, employment status, educational level, smoking and alcohol drinking status, reimbursement scheme, living distance from PD center, urgent-start PD, estimated glomerular filtration rate at PD initiation, PD modality, etiology of end-stage kidney disease, Charlson comorbidity index, and residual kidney function, and laboratory results (serum creatinine, serum urea nitrogen, albumin, hemoglobin, ferritin, transferrin saturation, sodium, potassium, bicarbonate, calcium, phosphorus, intact parathyroid hormone, and alkaline phosphatase). Abbreviations: CI, confidence interval; HR, hazard ratio; PD, peritoneal dialysis; SHR, subdistribution hazard ratio.

No collinearity was identified in the final model of analysis (Table S2 in the Supplementary Materials). Schoenfeld residual-based tests held the proportional hazards assumption for both all-cause mortality and cardiovascular death outcomes. Using shared frailty correction analysis, individuals experienced with any PD-associated peritonitis were associated with a higher risk of both all-cause mortality (full model adjusted HR, 2.17; 95% CI, 1.78–2.66; *p* < 0.001) and cardiovascular death (full model adjusted HR, 2.90; 95% CI, 2.05–4.59; *p* < 0.001) in all 3 models of adjustments (Figure 3 and Table S3 in the Supplementary Materials). These were consistent based on competing risk analysis: full model adjusted SHR, 2.00 (95% CI, 1.74–2.29; *p* < 0.001) for all-cause mortality and full model adjusted SHR, 2.25 (95% CI, 1.66–3.05; *p* < 0.001) for cardiovascular death (Figure 3 and Table S3 in the Supplementary Materials).

### 3.3. Further Analyses

With respect to subgroup analyses (Table 2), no additional effects based on age, sex, PD modality, and comorbid conditions were observed (all *p* for interaction >0.05). For the additional analysis, with a wide range of effect estimates, we also found that individuals with only 1 or ≥2 episodes and those who had early-onset or late-onset of PD-associated peritonitis were at risk of both all-cause mortality (full model adjusted HRs or SHRs ranging from 1.30 to 5.43) and cardiovascular death (full model adjusted HRs or SHRs ranging from 1.28 to 5.69) compared to individuals without PD-associated peritonitis (Table S4 in the Supplementary Materials).

In the sensitivity analyses without shared frailty correction, the results were also consistent with the main findings for both all-cause mortality and cardiovascular death outcomes (Table S5 in the Supplementary Materials). Based on the E-value estimation (Table S6 and Figure S1 in the Supplementary Materials), an unmeasured confounder could fully account for the association between PD-associated peritonitis and all-cause mortality. However, the point estimates of E-value and the lower limit of the 95% CI suggest that residual confounders are unlikely to fully explain the association between PD-associated peritonitis and cardiovascular death.

## 4. Discussion

### 4.1. Principal Findings

In this retrospective time-matched cohort of PD patients, we found that individuals with any PD-associated peritonitis are at approximately 2-fold risk of all-cause mortality and cardiovascular death compared with matched individuals without PD-associated peritonitis during the follow-up treatment period. These associations were robust to several analysis approaches and tests of bias and confounding, supporting the impacts of PD-associated peritonitis on long-term mortality among ESKD patients undergoing PD. However, our findings suggest that risk estimates for cardiovascular death appear to be influenced by unmeasured confounders.

### 4.2. Comparison with the Relevant Evidence

Collectively, with limited relevant clinical variables for adjustment, our findings are in line with those results from single-center [20] and local or national registries [10,11,18]. These studies found that PD-associated peritonitis was significantly associated with all-cause mortality (HR ranged from 1.95 to 2.01) [18,20] and cardiovascular death (HR ranged from 1.22 to 3.84) [10,11,20], which is supported by our findings. Considering different methodological approaches, we expanded on previous studies by accounting for a time-lead bias and competing risk of death in analyses and using patient-level data based on various modelling approaches to address confounders. This approach enables our methods of analysis to provide more precise information on the risk of death and PD-associated peritonitis.

Although we found a consistent finding based on different modelling analyses, the pathogenesis and mechanism underlying the long-term risk of mortality following PD-associated peritonitis are not well explained. Apart from acute infectious-related mortality, existing literature has hypothesized that alterations and deteriorations in residual kidney function and peritoneal membrane function following peritonitis over time may contribute directly to further long-term mortality risk [29]. Furthermore, systemic chronic inflammation cascades following a peritonitis episode may also contribute to cardiovascular events and mortality risks [30]. Given the complex interrelationship, we postulated that the association between PD-associated peritonitis and mortality risk may exhibit an interplay of both local and systemic peritoneal-kidney chronic inflammation in addition to comorbid conditions.

### 4.3. Considerations for Practice and Future Research

Randomized clinical trials to directly evaluate the long-term mortality impact of PD-associated peritonitis are unlikely to be feasible. In this context, our findings provide robust evidence, supported by consistent findings across multiple modeling approaches, that PD-associated peritonitis is significantly associated with increased risks of both all-cause and cardiovascular mortality. These findings have important implications for the design of sustainable PD programs, underscoring the urgent need for strategies that extend beyond infection control to include post-peritonitis risk mitigation and cardiovascular prevention. While several pharmacological and non-pharmacological strategies for the prevention of PD-related infections are currently under investigation, few studies have directly evaluated their effects on long-term mortality or adverse cardiovascular outcomes [16,17]. Future clinical trials should prioritize the inclusion of diverse PD populations and consider long-term mortality as a part of the core outcomes set. Moreover, further research into the pathophysiologic mechanisms linking peritonitis to downstream cardiovascular and mortality risks may help identify novel therapeutic targets. A deeper understanding of host immune responses, inflammation pathways, and the cumulative burden of infection-related insults may inform more comprehensive models of care for PD patients at heightened risk.

### 4.4. Strengths and Limitations

Based on a time-matched pairs analysis 1:1, we minimized bias by adjusting a series of important sociodemographic and clinical variables, rigorous exposure and outcomes ascertainment based on international guidelines, and medical chart review, having a long-period and complete follow-up using a patient-level database, accounting for the competing risk of death, and estimating the effect of unmeasured confounders in our analyses. This approach helped to overcome methodological limitations of the previous literature.

However, several limitations must be acknowledged. First, this study was fundamentally limited by its inability to determine causality due to the observational, retrospective cohort design. Second, despite controlling for multiple relevant confounders, the estimated E-value suggested that unmeasured confounders could not be fully accounted for, particularly the effect estimation of cardiovascular death. However, risk estimates using shared frailty correction and competing risk analyses, based on a series of degree confounder adjustments, were robust, indicating limited residual confounders in our findings. Third, inflammatory mediators, such as nutritional status, peritoneal membrane function, and relevant chronic inflammatory markers, as well as compelling cardiovascular and antihypertensive medications, were not available in this cohort, which might underpin the pathogenesis of cardiovascular events and the excess mortality following a peritonitis episode. However, several surrogate variables were used instead, such as serum albumin and ferritin level, to account for this issue. Fourth, investigating the impact of severity strata according to organism-specific peritonitis should be considered; however, it is beyond the design of the present study. Moreover, based on additional analyses, we did not find any significant dose–response relationships or increased mortality risk associated with the frequency or onset of PD-associated peritonitis. Fifth, our study relied on ESKD patients who commenced KRT under the “PD First” policy, which represents the majority of CAPD patients in Thailand, and had limited racial and ethnic diversity. Although PD treatment practices, such as strategies for the prevention and management of catheter-related infections, as well as approaches for management of cardiovascular risk factors, were not substantially different across the PD centers in this study. However, treatment practices (e.g., details for teaching and retraining PD programs to patients and caregivers, and strategies for nutritional promotion) and medication optimization for the prevention of peritoneal membrane dysfunction may not be uniform over time, which may have influenced the findings. Moreover, our findings relied on data from large PD centers, which likely had more PD practice experience in managing a higher volume of PD cases compared to other facilities. As a result, our findings may limit generalizability to broader PD populations or modalities (i.e., APD), particularly in different health care resources or settings.

## 5. Conclusions

In this time-matched retrospective cohort study, individuals who experienced PD-associated peritonitis had significantly higher risks of all-cause and cardiovascular mortality compared to PD patients without peritonitis. These associations remained robust across multiple risk-modeling approaches. To promote sustainable long-term PD treatment, future research should prioritize the development and evaluation of targeted interventions aimed at reducing mortality risk following peritonitis.

## Figures and Tables

**Table 1:** Patient Characteristics Based on a Time-Matched Cohort (1:1) ^†^.

Characteristics	Overall (n = 3020)	Without Peritonitis (n = 1510)	With Any PD-Associated Peritonitis (n = 1510)	*p*-Value
**Matching variables**				
Age, years	58.6 ± 14.2	58.6 ± 14.2	58.5 ± 14.2	0.962
<65	2007 (66.5)	1010 (66.9)	997 (66.0)	0.644
≥65	1013 (33.5)	500 (33.1)	513 (34.0)	
Male	1618 (53.6)	809 (53.6)	809 (53.6)	1.000
Time-to-index date, month; median (min–max) (time-matched) ^‡^	10.0 (0.1–86.4)	10.0 (0.1–86.4)	10.0 (0.1–86.4)	0.958
**Sociodemographic**				
BMI, kg/m^2^	21.4 ± 3.6	21.3 ± 3.3	21.6 ± 3.8	0.028
<18.5	644 (21.3)	316 (20.9)	328 (21.7)	0.008
18.5–22.9	1519 (50.3)	799 (52.9)	720 (47.7)	
≥23.0	857 (28.4)	395 (26.2)	462 (30.6)	
Marital status				
Single	411 (13.6)	169 (11.2)	242 (16.0)	<0.001
Married	2154 (71.3)	1115 (73.8)	1039 (68.8)	
Widow/divorced/other	455 (15.1)	226 (15.0)	229 (15.2)	
Unemployed/retried	886 (29.3)	461 (30.5)	425 (28.2)	0.162
Educational level				
Illiterate/primary school	2146 (71.1)	1024 (67.8)	1122 (74.3)	<0.001
Junior to high school	421 (13.9)	198 (13.1)	223 (14.8)	
Diploma/bachelor/higher	453 (15.0)	288 (19.1)	165 (10.9)	
Current smoker ^§^	175 (5.8)	61 (4.0)	114 (7.6)	<0.001
Current alcohol drinking ^§^	211 (7.0)	100 (6.6)	111 (7.4)	0.475
Reimbursement scheme				
NHSO	2089 (69.2)	980 (64.9)	1109 (73.4)	<0.001
CSMB	737 (24.4)	503 (33.3)	234 (15.5)	
Others	194 (6.4)	27 (1.8)	167 (11.1)	
Living distance ≥100 km from PD center	674 (22.3)	355 (23.5)	319 (21.1)	0.126
**PD and clinical characteristics**				
Era of PD initiation				
2006–2010	729 (24.1)	300 (19.9)	429 (28.4)	<0.001
2011–2015	1536 (50.9)	760 (50.3)	776 (51.4)	
2016–2020	755 (25.0)	450 (29.8)	305 (20.2)	
Urgent-start PD based on catheter break-in time ≤14 days	393 (13.0)	112 (7.4)	281 (18.6)	<0.001
eGFR at PD initiation, mL/min/1.73 m^2^	6.1 ± 3.0	6.4 ± 3.1	5.7 ± 2.9	<0.001
Early start (≥10)	404 (13.4)	247 (16.4)	157 (10.4)	<0.001
Intermediate start (5–9.9)	1241 (41.1)	640 (42.4)	601 (39.8)	
Late start (<5)	1375 (45.5)	623 (41.2)	752 (49.8)	
PD modality				
APD	405 (13.4)	264 (17.5)	141 (9.3)	<0.001
CAPD	2615 (86.6)	1246 (82.5)	1369 (90.7)	
Etiology of ESKD				
Hypertensive nephrosclerosis	1068 (35.4)	566 (37.5)	502 (33.2)	0.001
Diabetic nephropathy	1065 (35.3)	479 (31.7)	586 (38.8)	
Glomerulonephritis	275 (9.1)	142 (9.4)	133 (8.8)	
Unknown/others	612 (20.3)	323 (21.4)	289 (19.1)	
Charlson comorbidity index; median (min–max)	5 (2–14)	5 (2–14)	6 (2–14)	0.020
Low (<4)	583 (19.3)	299 (19.8)	284 (18.8)	0.002
Medium (4–6)	1517 (50.2)	795 (52.6)	722 (47.8)	
High (>6)	920 (30.5)	416 (27.6)	504 (33.4)	
Comorbid conditions				
Coronary artery disease	562 (18.6)	229 (15.2)	333 (22.1)	<0.001
Chronic heart failure	385 (12.8)	138 (9.1)	247 (16.4)	<0.001
Cerebrovascular disease	445 (14.7)	139 (9.2)	306 (20.3)	<0.001
Diabetes mellitus	1220 (40.4)	544 (36.0)	676 (44.8)	<0.001
Residual kidney function (urine output ≥200 mL/day)	2160 (71.5)	1174 (77.8)	986 (65.3)	<0.001
Onset of PD-related peritonitis				
Early-onset (time-to-first episode ≤3 months)	286 (9.5)	NA	286 (18.9)	NA
Late-onset (time-to-first episode >3 months)	1224 (40.5)	NA	1224 (81.1)	NA
No. of PD-related peritonitis episodes; median (min–max)	0.5 (0–10)	NA	2 (1–10)	NA
**Laboratory profiles**				
Creatinine, mg/dL	10.1 ± 4.1	10.2 ± 4.2	10.0 ± 4.1	0.313
Urea nitrogen, mg/dL	59.5 ± 18.6	56.6 ± 17.7	62.5 ± 19.0	<0.001
Serum albumin, g/dL	3.4 ± 0.6	3.7 ± 0.5	3.1 ± 0.5	<0.001
Hemoglobin, g/dL	9.2 ± 1.2	9.6 ± 1.1	8.9 ± 1.2	<0.001
Ferritin, ng/mL; median (min–max)	591 (25.5–2889)	583 (25.5–2359.5)	602 (37–2889)	0.063
TSAT, %	25.9 ± 4.4	25.8 ± 3.6	26.1 ± 5.0	0.098
Sodium, mEq/L	136.2 ± 3.9	138.0 ± 3.6	134.5 ± 3.5	<0.001
Potassium, mEq/L	3.8 ± 0.7	4.2 ± 0.7	3.4 ± 0.5	<0.001
Bicarbonate, mEq/L	27.4 ± 4.1	27.9 ± 4.0	26.9 ± 4.1	<0.001
Calcium, mg/dL	8.5 ± 1.2	8.6 ± 1.2	8.5 ± 1.2	0.011
Phosphorus, mg/dL	3.9 ± 1.4	4.4 ± 1.2	3.5 ± 1.4	<0.001
iPTH, pg/mL; median (min–max)	414 (17.2–2065)	412.5 (25–1922)	416.2 (17.2–2065)	0.802
ALP, U/L; median (min–max)	107 (21–443)	87 (21–443)	127 (32–433)	<0.001

^†^ Categorical variables are expressed as percentage; continuous variables as mean ± standard deviation, unless otherwise indicated. ^‡^ Time from PD initiation to the occurrence of the first episode of PD-related peritonitis was matched with individuals without any PD-related peritonitis. ^§^ Defined as those who were current smokers (drinkers) or smokers (drinkers) for at least 6 months in the past and quit smoking (drinking) less than 12 months. Abbreviations: ALP, alkaline phosphatase; APD, automated peritoneal dialysis; BMI, body mass index; CAPD, continuous ambulatory peritoneal dialysis; CSMB, Civil Servant Medical Benefit; eGFR, estimated glomerular filtration rate; ESKD, end-stage kidney disease; iPTH, intact parathyroid hormone; NA, not applicable; NHSO, National Health Security Office; PD, peritoneal dialysis; TSAT, transferrin saturation.

**Table 2:** Subgroup Analyses of PD-Associated Peritonitis and Subsequent Risk of All-Cause Mortality and Cardiovascular Death ^†^.

Subgroup Analysis	All-Cause Mortality						Cardiovascular Death					
	Death, No. (%)		Cox Proportional Hazards Model with Shared Frailty Correction Analysis: HR (95% CI)	*p* for Interaction	Competing Risk Analysis: SHR (95% CI)	*p* for Interaction	Death, No. (%)		Cox Proportional Hazards Model with Shared Frailty Correction Analysis: HR (95% CI)	*p* for Interaction	Competing Risk Analysis: SHR (95% CI)	*p* for Interaction
	Individuals Without PD-Associated Peritonitis	Individuals with Any PD-Associated Peritonitis					Individuals Without PD-Associated Peritonitis	Individuals with Any PD-Associated Peritonitis				
Age, years												
<65	130 (32.8)	267 (67.2)	2.07 (1.39–3.10)	0.258	2.19 (1.73–2.78)	0.204	30 (13.9)	186 (86.1)	1.60 (1.02–3.71)	0.644	1.61 (1.25–2.80)	0.301
≥65	420 (46.8)	477 (53.2)	3.48 (2.61–4.64)		2.03 (1.73–2.37)		90 (32.4)	188 (67.6)	2.81 (1.29–4.35)		2.54 (1.76–3.67)	
Sex												
Male	348 (43.8)	447 (56.2)	1.76 (1.37–2.28)	0.221	1.93 (1.63–2.30)	0.570	72 (25.4)	212 (74.6)	2.91 (1.21–6.35)	0.516	1.69 (1.16–2.46)	0.874
Female	202 (40.1)	297 (59.5)	2.30 (1.61–3.28)		2.26 (1.82–2.85)		48 (22.9)	162 (77.1)	3.56 (1.47–6.41)		2.40 (1.44–4.00)	
PD modality												
APD	136 (62.4)	82 (37.6)	1.72 (1.05–2.82)	0.509	1.49 (1.04–2.15)	0.187	34 (50.8)	33 (49.2)	2.43 (1.21–5.82)	0.809	2.54 (1.08–5.98)	0.391
CAPD	414 (38.5)	662 (61.5)	1.89 (1.49–2.39)		2.03 (1.75–2.36)		86 (20.1)	341 (79.9)	2.19 (1.83–5.71)		1.91 (1.36–2.69)	
History of Coronary artery disease												
No	326 (44.0)	414 (56.0)	2.31 (1.76–3.04)	0.424	2.29 (1.92–2.72)	0.092	78 (24.8)	237 (75.2)	3.15 (1.51–7.32)	0.660	2.17 (1.47–3.19)	0.326
Yes	224 (40.4)	330 (59.6)	2.19 (1.45–3.32)		1.61 (1.32–1.96)		42 (23.5)	137 (76.5)	2.62 (1.17–5.87)		1.99 (1.22–3.26)	
History of chronic heart failure												
No	414 (45.4)	499 (54.6)	2.02 (1.59–2.56)	0.448	1.99 (1.70–2.32)	0.958	91 (25.0)	273 (75.0)	3.53 (1.88–7.63)	0.784	2.00 (1.41–2.84)	0.411
Yes	136 (35.7)	245 (64.3)	3.24 (1.85–5.69)		2.26 (1.78–2.87)		29 (22.3)	101 (77.7)	3.38 (1.99–7.40)		1.88 (1.23–3.83)	
History of cerebrovascular disease												
No	414 (48.3)	443 (51.7)	1.99 (1.55–2.56)	0.227	1.94 (1.64–2.28)	0.391	90 (26.4)	251 (73.6)	2.07 (1.92–6.39)	0.724	1.86 (1.30–2.66)	0.518
Yes	136 (31.1)	301 (68.9)	2.76 (1.73–4.42)		1.60 (1.26–2.03)		30 (19.6)	123 (80.4)	2.39 (1.60–6.08)		2.25 (1.24–4.10)	
History of diabetes mellitus												
No	201 (47.0)	227 (53.0)	2.15 (1.45–3.18)	0.203	2.76 (2.00–3.50)	0.113	37 (21.5)	135 (78.5)	5.15 (2.68–9.90)	0.577	2.04 (1.14–3.66)	0.520
Yes	349 (40.3)	517 (59.7)	1.80 (1.39–2.34)		1.81 (1.53–2.15)		83 (25.8)	239 (74.2)	4.26 (2.62–6.94)		1.89 (1.30–2.76)	

^†^ Based on adjusted model 3 (full model) includes: year of PD initiation and center of PD treatment, age, sex, body mass index, marital status, employment status, educational level, smoking and alcohol drinking status, reimbursement scheme, living distance from PD center, urgent-start PD, estimated glomerular filtration rate at PD initiation, PD modality, etiology of end-stage kidney disease, Charlson comorbidity index, and residual kidney function, and laboratory results (serum creatinine, serum urea nitrogen, albumin, hemoglobin, ferritin, transferrin saturation, sodium, potassium, bicarbonate, calcium, phosphorus, intact parathyroid hormone, and alkaline phosphatase). Abbreviations: CI, confidence interval; HR, hazard ratio; PD, peritoneal dialysis; SHR, subdistribution hazard ratio.

## Data Availability

The data underlying this article are not publicly available due to privacy and ethical restrictions. Data will be shared upon reasonable request and with permission according to the Thai Renal Outcomes Research-Peritoneal Dialysis (THOR-PD) Research Group data release policy and signing of a data transfer agreement.

## Supplementary Materials

The following supporting information can be downloaded at: https://www.mdpi.com/article/10.3390/medsci13040249/s1, Table S1: Crude Rates of All-Cause Mortality and Cardiovascular Death Following PD-Associated Peritonitis; Table S2: Collinearity Diagnostics; Table S3: PD-Associated Peritonitis and Mortality Outcomes; Table S4: Additional Analysis; Table S5: Sensitivity Analysis: Cox Proportional Hazards Model Without Shared Frailty Correction Analysis; Table S6: Sensitivity Analysis: E-Value; Figure S1: Estimation of E-Value.
