# Supplementary material for: Association Between Peritoneal Dialysis-Associated Peritonitis and the Risk of All-Cause Mortality and Cardiovascular Death: A Time-Matched Retrospective Cohort Study
Source: Med Sci (Basel). 2025 Oct 30;13(4):249. doi: 10.3390/medsci13040249 (PMC12641846; doi:10.3390/medsci13040249)

Supplementary Materials

Peritoneal Dialysis-Associated Peritonitis and Risk of All-Cause Mortality and Cardiovascular Death: A Time-Matched Retrospective Cohort Study

Surapon Nochaiwong, Kajohnsak Noppakun, Manish M. Sood, Kednapa Thavorn, Greg A. Knoll, Chidchanok Ruengorn, Apichat Tantraworasin

Supplementary Online Content

**Table S1**	Crude Rates of All-Cause Mortality and Cardiovascular Death Following PD-Associated Peritonitis	S3
**Table S2**	Collinearity Diagnostics	S4
**Table S3**	PD-Associated Peritonitis and Mortality Outcomes	S5
**Table S4**	Additional Analysis	S6
**Table S5**	Sensitivity Analysis: Cox Proportional Hazards Model Without Shared Frailty Correction Analysis	S7
**Table S6**	Sensitivity Analysis: E-Value	S8
**Figure S1**	Estimation of E-Value	S9

Table S1 Crude Rates of All-Cause Mortality and Cardiovascular Death Following PD-Associated Peritonitis

**Outcomes**	**All-cause mortality**			**Cardiovascular death**		
	**Overall**	**Individuals without PD-associated peritonitis**	**Individuals with any PD-associated peritonitis**	**Overall**	**Individuals without PD-associated peritonitis**	**Individuals with any PD-associated peritonitis**
No. of death (%)/total sample	1,294 (42.8)/3,020	550 (36.4)/1,510	744 (49.3)/1,510	494 (16.4%)/3020	120 (8.0%)/1510	374 (24.8%)/1510
Patient-years of follow-up	11,064	6,874.1	4,189.9	11,064	6,874.1	4,189.9
Median time survival, year (95% CI)	5.6 (5.2 – 6.2)	10.0 (9.1 – 10.3)	4.1 (3.8 – 4.4)	NA (NA)	NA (NA)	6.1 (5.6 – 6.6)
Incidence rate, per 100 patient-years (95% CI)	11.7 (11.1 – 12.4)	8.0 (7.4 – 8.7)	17.8 (16.5 – 19.1)	4.5 (4.1 – 4.9)	1.7 (1.4 – 2.1)	8.9 (8.1 – 9.9)
Absolute risk difference (crude %)		12.9 (8.2 – 17.7)			18.6 (14.8 – 22.3)	

Abbreviations: CI, confidence interval; NA, not applicable; PD, peritoneal dialysis.

Table S2 Collinearity Diagnostics

Variables	VIF†	Tolerance‡
Year of PD initiation	1.07	0.93
Center of PD treatment	1.02	0.98
Age, years	1.95	0.51
Sex	1.08	0.92
BMI, kg/m^2	1.04	0.96
Marital status	1.24	0.80
Unemployed/retried	1.30	0.77
Educational level	1.30	0.77
Current smoker	1.31	0.76
Current alcohol drinking	1.47	0.68
Reimbursement scheme	1.25	0.80
Living distance ≥100 km from PD center	1.03	0.97
Urgent-start PD based on catheter break-in time	1.33	0.75
eGFR at PD initiation, mL/min/1.73 m^2	1.12	0.90
PD modality	1.29	0.77
Etiology of ESKD	1.04	0.96
Charlson comorbidity index	1.99	0.50
RKF (urine output ≥200 mL/day)	1.25	0.80
Creatinine, mg/dL	1.18	0.85
SUN, mg/dL	1.17	0.86
Serum albumin, g/dL	1.67	0.60
Hemoglobin, g/dL	1.61	0.62
Ferritin, ng/mL	1.21	0.82
TSAT, %	1.11	0.90
Sodium, mEq/L	1.03	0.97
Potassium, mEq/L	1.44	0.70
Bicarbonate, mEq/L	1.32	0.76
Calcium, mg/dL	1.21	0.83
Phosphorus, mg/dL	1.46	0.69
iPTH, pg/mL	1.23	0.81
ALP, U/L	1.66	0.60
Mean VIF	1.30	

Abbreviations: ALP, alkaline phosphatase; BMI, body mass index; eGFR, estimated glomerular filtration rate; ESKD, end-stage kidney disease; iPTH, intact parathyroid hormone; PD, peritoneal dialysis; RKF, residual kidney function; SUN, serum urea nitrogen; TSAT, transferrin saturation; VIF, variance inflation factor.

†VIF <2.5 indicates lack of collinearity.

‡Tolerance is the reciprocal of VIF, with a value of 0.40 or less is cause for concern.

Table S3 PD-Associated Peritonitis and Mortality Outcomes

Mortality outcomes	All-cause mortality				Cardiovascular death			
	Cox proportional hazards model with shared frailty correction analysis: HR (95% CI)	*P* value	Competing risk analysis: SHR (95% CI)	*P* value	Cox proportional hazards model with shared frailty correction analysis: HR (95% CI)	*P* value	Competing risk analysis: SHR (95% CI)	*P* value
**Main analysis**								
Unadjusted	18.06 (15.04 – 21.67)	<0.001	1.61 (1.44 – 1.79)	<0.001	24.70 (18.40 – 33.16)	<0.001	3.45 (2.81 – 4.23)	<0.001
Adjusted model 1[a]	18.18 (15.11 – 21.88)	<0.001	1.69 (1.51 – 1.89)	<0.001	26.44 (19.49 – 35.87)	<0.001	3.56 (2.90 – 4.36)	<0.001
Adjusted model 2[b]	5.64 (4.80 – 6.63)	<0.001	3.72 (2.76 – 5.23)	0.003	16.45 (12.21 – 22.16)	<0.001	3.46 (2.77 – 4.32)	<0.001
Adjusted model 3 (full model)[c]	2.17 (1.78 – 2.66)	<0.001	2.00 (1.74 – 2.29)	<0.001	2.90 (2.05 – 4.59)	<0.001	2.25 (1.66 – 3.05)	<0.001

[a]Adjusted model 1 includes: year of PD initiation and center of PD treatment.
[b]Adjusted model 2 includes: model 1 plus age, sex, body mass index, marital status, employment status, educational level, smoking and alcohol drinking status, reimbursement scheme, living distance from PD center, urgent-start PD, eGFR at PD initiation, PD modality, etiology of ESKD, Charlson comorbidity index, and residual kidney function.
[c]Adjusted model 3 includes: model 2 plus laboratory results (serum creatinine, serum urea nitrogen, albumin, hemoglobin, ferritin, transferrin saturation, sodium, potassium, bicarbonate, calcium, phosphorus, intact parathyroid hormone, and alkaline phosphatase).
Abbreviations: CI, confidence interval; eGFR, estimated glomerular filtration rate; ESKD, end-stage kidney disease; HR, hazard ratio; PD, peritoneal dialysis; SHR, subdistribution hazard ratio.

Table S4 Additional Analysis

Mortality outcomes (reference group: individuals without PD-associated peritonitis)	All-cause mortality				Cardiovascular death			
	Cox proportional hazards model with shared frailty correction analysis: HR (95% CI)	*P* value	Competing risk analysis: SHR (95% CI)	*P* value	Cox proportional hazards model with shared frailty correction analysis: HR (95% CI)	*P* value	Competing risk analysis: SHR (95% CI)	*P* value
**No. of PD-associated peritonitis episodes**								
• **1 episode**								
Unadjusted	63.16 (45.83 – 87.04)	<0.001	1.32 (1.11 – 1.56)	0.001	29.86 (19.31 – 46.18)	<0.001	3.28 (2.52 – 4.28)	<0.001
Adjusted model 1[a]	63.32 (45.94 – 87.27)	<0.001	1.35 (1.14 – 1.60)	0.001	31.54 (20.31 – 48.97)	<0.001	3.38 (2.59 – 4.42)	<0.001
Adjusted model 2[b]	14.39 (11.13 – 18.60)	<0.001	2.82 (2.23 – 3.56)	<0.001	37.46 (24.54 – 57.18)	<0.001	6.22 (4.56 – 8.49)	<0.001
Adjusted model 3 (full model)[c]	5.43 (2.07 – 7.23)	<0.001	1.49 (1.17 – 1.90)	0.001	5.69 (3.68 – 9.43)	<0.001	2.93 (2.04 – 4.20)	<0.001
• **≥2 episode**s								
Unadjusted	16.43 (13.55 – 19.91)	<0.001	1.73 (1.54 – 1.95)	<0.001	23.92 (17.38 – 32.93)	<0.001	3.95 (3.20 – 4.89)	<0.001
Adjusted model 1[a]	16.38 (13.48 – 19.92)	<0.001	1.85 (1.64 – 2.08)	<0.001	25.60 (18.39 – 35.65)	<0.001	4.22 (3.40 – 5.24)	<0.001
Adjusted model 2[b]	4.56 (3.85 – 5.39)	<0.001	1.87 (1.62 – 2.16)	<0.001	13.28 (9.76 – 18.07)	<0.001	4.20 (3.33 – 5.30)	<0.001
Adjusted model 3 (full model)[c]	1.64 (1.32 – 2.72)	<0.001	1.82 (1.71 – 2.31)	0.003	4.74 (3.22 – 6.96)	<0.001	1.72 (1.26 – 2.35)	0.001
**Onset of PD-associated peritonitis**								
• **Early-onset (time-to-first episode ≤3 months)**								
Unadjusted	13.91 (10.46 – 18.50)	<0.001	1.90 (1.58 – 2.27)	<0.001	23.16 (14.38 – 37.30)	<0.001	3.94 (2.95 – 5.27)	<0.001
Adjusted model 1[a]	13.96 (10.47 – 18.61)	<0.001	1.93 (1.61 – 2.32)	<0.001	24.28 (14.94 – 39.46)	<0.001	4.00 (2.99 – 5.34)	<0.001
Adjusted model 2[b]	3.86 (2.92 – 5.11)	<0.001	1.81 (1.44 – 2.27)	<0.001	9.99 (6.18 – 16.16)	<0.001	3.60 (2.52 – 5.14)	<0.001
Adjusted model 3 (full model)[c]	2.22 (1.89 – 3.60)	0.018	1.30 (1.15 – 1.89)	0.042	2.82 (1.63 – 4.89)	<0.001	1.28 (1.03 – 1.94)	0.040
• **Late-onset (time-to-first episode >3 months)**								
Unadjusted	19.88 (16.32 – 24.23)	<0.001	1.54 (1.37 – 1.73)	<0.001	25.23 (18.50 – 34.41)	<0.001	3.71 (3.01 – 4.58)	<0.001
Adjusted model 1[a]	20.01 (16.38 – 24.45)	<0.001	1.63 (1.45 – 1.83)	<0.001	27.17 (19.71 – 37.44)	<0.001	3.94 (3.19 – 4.88)	<0.001
Adjusted model 2[b]	5.89 (4.98 – 6.96)	<0.001	2.09 (1.81 – 2.41)	<0.001	181.19 (13.35 – 24.78)	<0.001	4.77 (3.81 – 5.98)	<0.001
Adjusted model 3 (full model)[c]	2.15 (1.75 – 2.65)	<0.001	2.10 (1.85 – 3.12)	<0.001	5.64 (3.54 – 8.71)	<0.001	2.09 (1.54 – 2.82)	<0.001

[a]Adjusted model 1 includes: year of PD initiation and center of PD treatment.
[b]Adjusted model 2 includes: model 1 plus age, sex, body mass index, marital status, employment status, educational level, smoking and alcohol drinking status, reimbursement scheme, living distance from PD center, urgent-start PD, eGFR at PD initiation, PD modality, etiology of ESKD, Charlson comorbidity index, and residual kidney function.
[c]Adjusted model 3 includes: model 2 plus laboratory results (serum creatinine, serum urea nitrogen, albumin, hemoglobin, ferritin, TSAT, sodium, potassium, bicarbonate, calcium, phosphorus, intact parathyroid hormone, and alkaline phosphatase).
Abbreviations: CI, confidence interval; HR, hazard ratio; PD, peritoneal dialysis; SHR, subdistribution hazard ratio.

Table S5 Sensitivity Analysis: Cox Proportional Hazards Model Without Shared Frailty Correction Analysis

**Mortality outcomes**	**All-cause mortality**		**Cardiovascular death**	
	**HR (95% CI)**	***P* value**	**HR (95% CI)**	***P* value**
**Main analysis**				
Unadjusted	2.24 (1.99 – 2.51)	<0.001	5.94 (4.77 – 7.40)	<0.001
Adjusted model 1[a]	2.36 (2.11 – 2.66)	<0.001	6.29 (5.04 – 7.86)	<0.001
Adjusted model 2[b]	2.98 (2.63 – 3.39)	<0.001	7.98 (6.29 – 10.12)	<0.001
Adjusted model 3 (full model)[c]	1.35 (1.16 – 1.58)	<0.001	3.07 (2.31 – 4.07)	<0.001

[a]Adjusted model 1 includes: year of PD initiation and center of PD treatment.

[b]Adjusted model 2 includes: model 1 plus age, sex, body mass index, marital status, employment status, educational level, smoking and alcohol drinking status, reimbursement scheme, living distance from PD center, urgent-start PD, eGFR at PD initiation, PD modality, etiology of ESKD, Charlson comorbidity index, and residual kidney function.

[c]Adjusted model 3 includes: model 2 plus laboratory results (serum creatinine, serum urea nitrogen, albumin, hemoglobin, ferritin, TSAT, sodium, potassium, bicarbonate, calcium, phosphorus, intact parathyroid hormone, and alkaline phosphatase).

Abbreviations: CI, confidence interval; HR, hazard ratio; PD, peritoneal dialysis.

Table S6 Sensitivity Analysis: E-Value[a]

Mortality outcomes	Cox proportional hazards model with shared frailty correction analysis: HR (95% CI)	*P* value	E-value for point estimates (95% CI lower limit)	Competing risk analysis: SHR (95% CI)	*P* value	E-value for point estimates (95% CI lower limit)
All-cause mortality	2.17 (1.78 – 2.66)	<0.001	3.763 (2.958)	2.00 (1.74 – 2.29)	<0.001	3.414 (2.875)
Cardiovascular death	2.90 (2.05 – 4.59)	<0.001	5.247 (3.517)	2.25 (1.66 – 3.05)	<0.001	3.927 (2.707)

[a]Based on adjusted model 3 (full model) includes: year of PD initiation and center of PD treatment, age, sex, body mass index, marital status, employment status, educational level, smoking and alcohol drinking status, reimbursement scheme, living distance from PD center, urgent-start PD, eGFR at PD initiation, PD modality, etiology of ESKD, Charlson comorbidity index, and residual kidney function, and laboratory results (serum creatinine, serum urea nitrogen, albumin, hemoglobin, ferritin, transferrin saturation, sodium, potassium, bicarbonate, calcium, phosphorus, intact parathyroid hormone, and alkaline phosphatase).

Abbreviations: CI, confidence interval; eGFR, estimated glomerular filtration rate; ESKD, end-stage kidney disease; HR, hazard ratio; PD, peritoneal dialysis; SHR, subdistribution hazard ratio.

Figure S1 Estimation of E-Value

A. HR for All-Cause Mortality

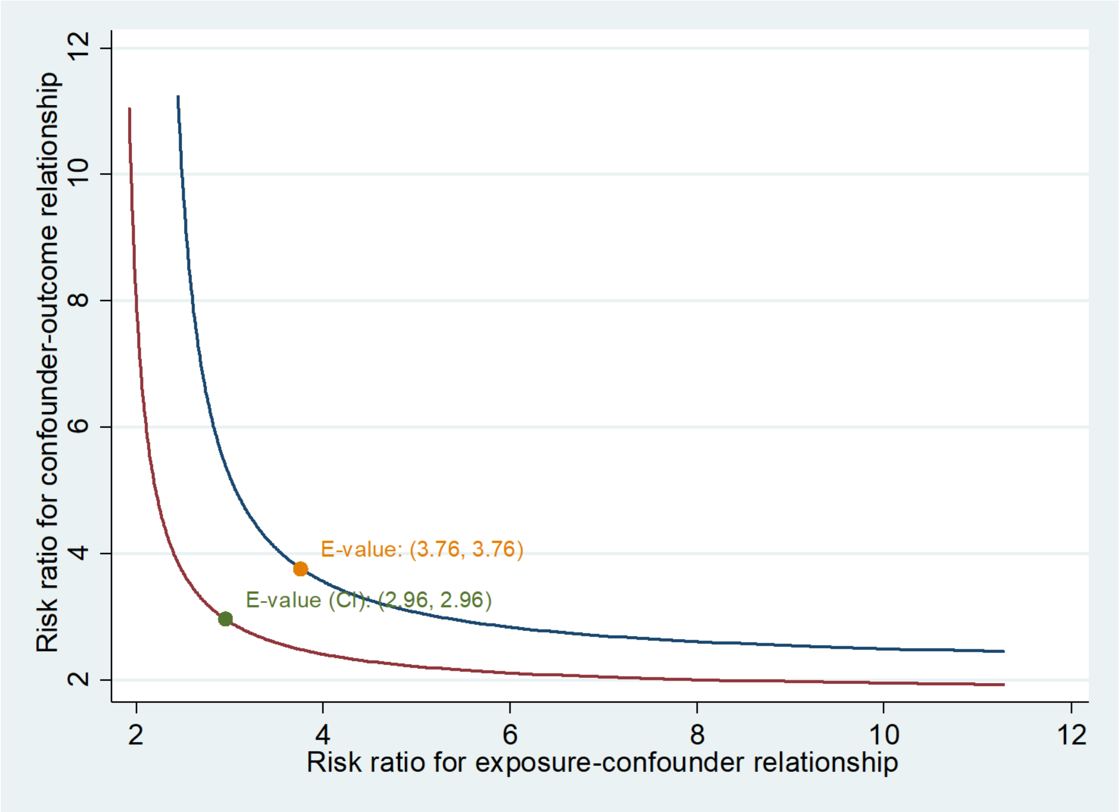

B. SHR for All-Cause Mortality

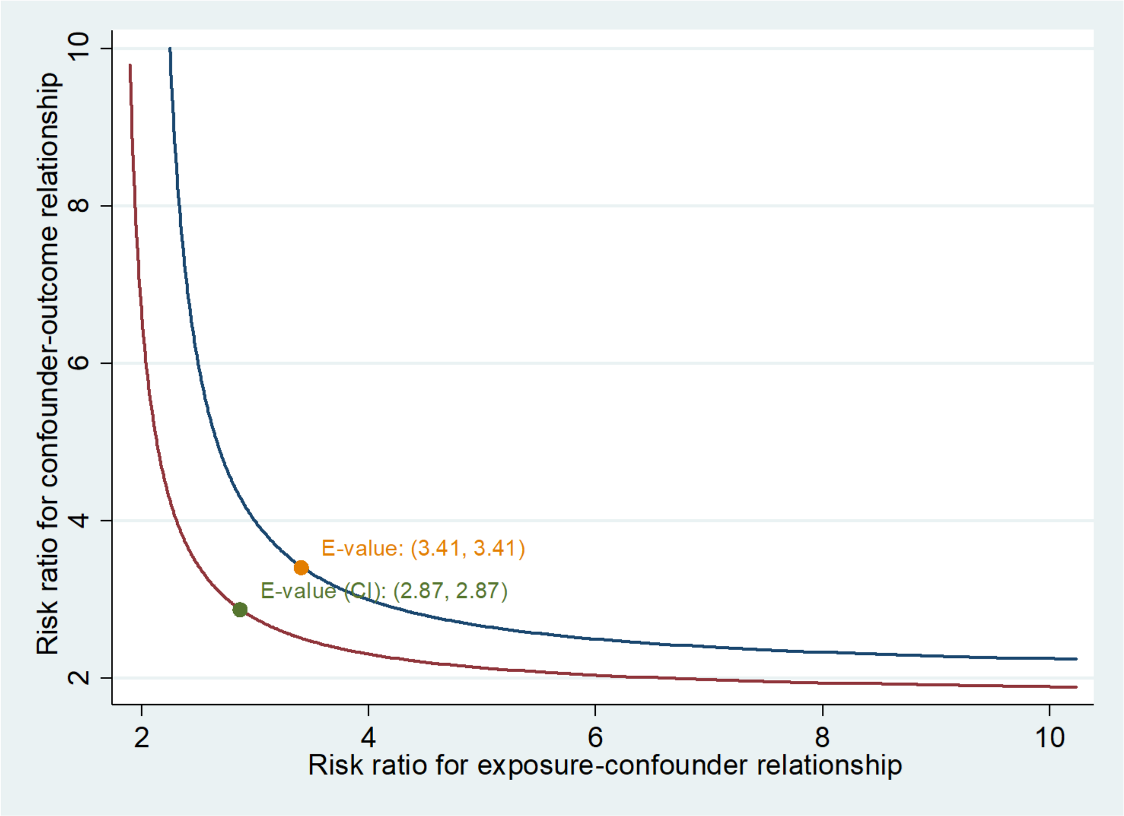

Figure S1 Estimation of E-Value (Continued)

C. HR for Cardiovascular Death

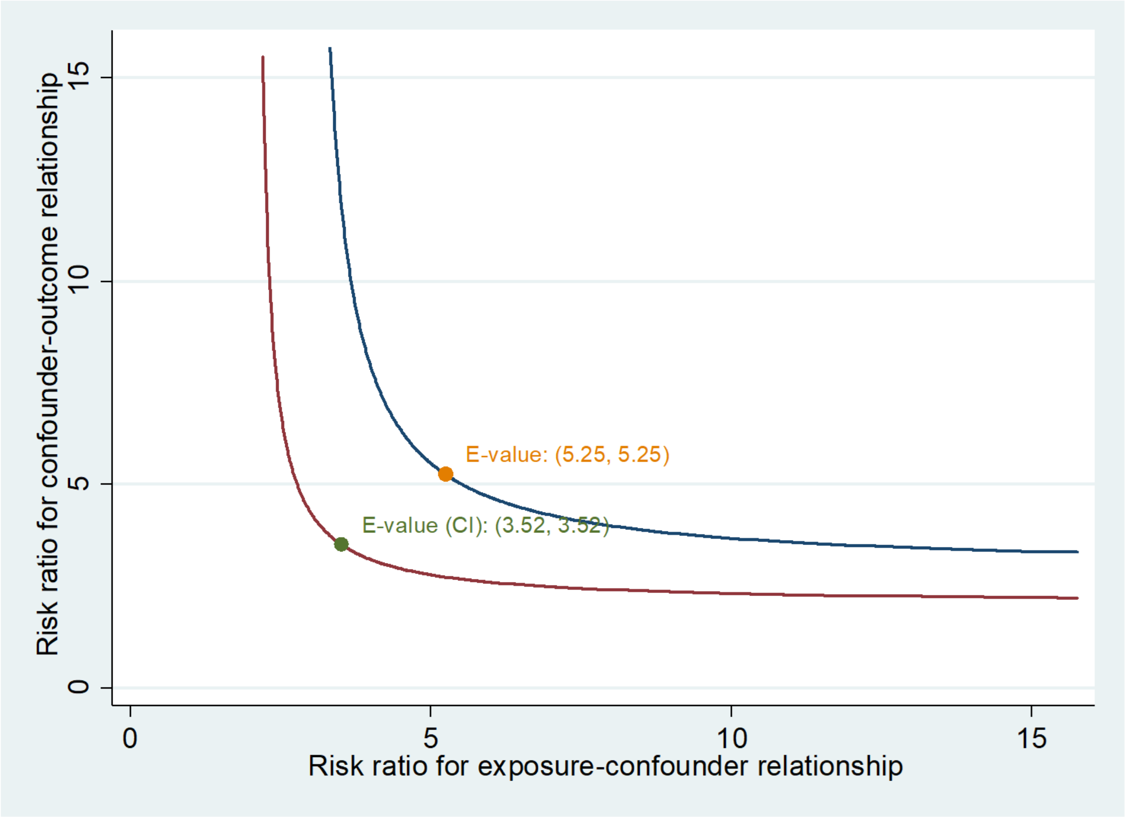

D. SHR for Cardiovascular Death

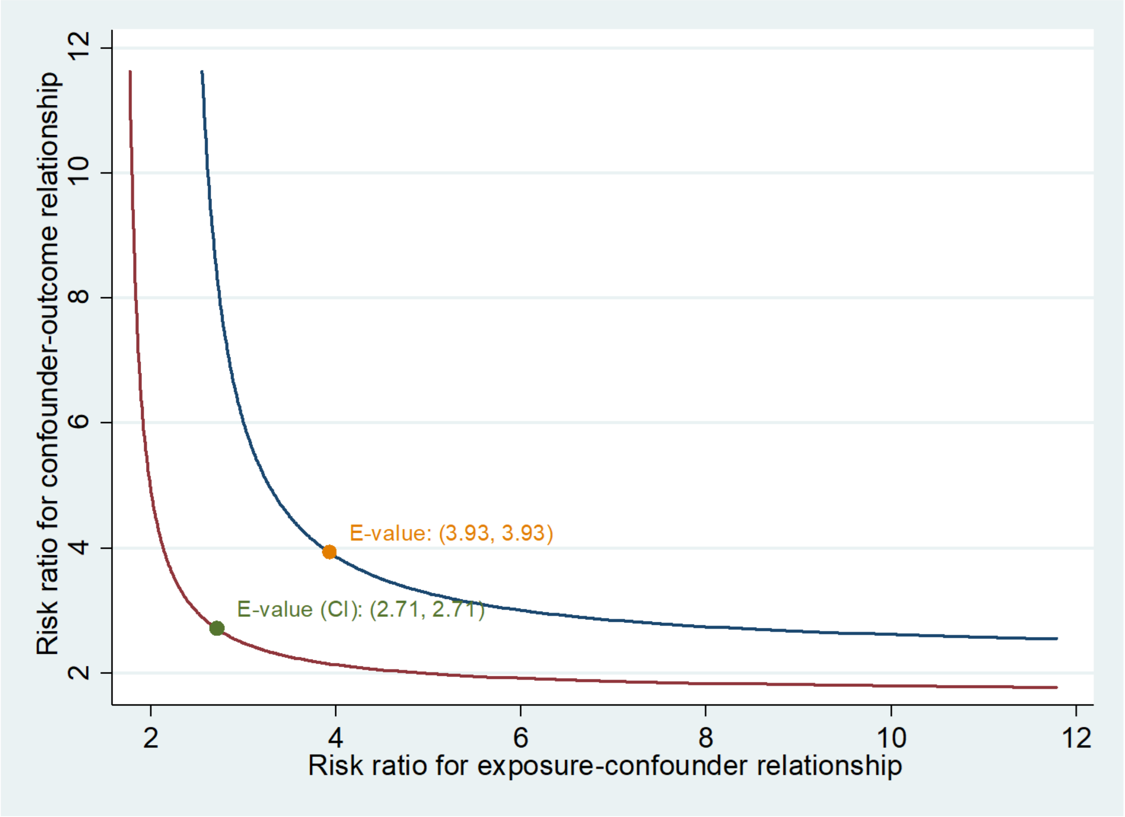

Supplement: Supplementary file 1 [file medsci-13-00249-s001.zip › medsci-3887141-supplementary.pdf]
